# Multi-modal sexual displays in Australian humpback dolphins

**DOI:** 10.1038/s41598-017-13898-9

**Published:** 2017-10-20

**Authors:** S. J. Allen, S. L. King, M. Krützen, A. M. Brown

**Affiliations:** 1 School of Biological Sciences and Oceans Institute, University of Western Australia, Crawley, Western Australia 6009 Australia; 2 Department of Anthropology, University of Zurich, CH-8057 Zurich, Switzerland; 3 Cetacean Research Unit, School of Veterinary and Life Sciences, Murdoch University, Murdoch, Western Australia, 6150 Australia; 4 Hartley Anderson Ltd, 36 Regent Quay, Aberdeen, AB11 5BE United Kingdom

## Abstract

Sexual displays enriched by object carrying serve to increase individual male fitness, yet are uncommon phenomena in the animal kingdom. While they have been documented in a variety of taxa, primarily birds, they are rare outside non-human mammals. Here, we document marine sponge presenting associated with visual and acoustic posturing found in several, geographically widespread populations of Australian humpback dolphins (*Sousa sahulensis*) over ten years of observation. Only adult males presented marine sponges, typically doing so in the presence of sexually mature females, although social groups predominantly consisted of mixed age and sex classes. Male humpback dolphins appear to be using sponges for signalling purposes in multi-modal sexual displays. Further, based on limited behavioural and genetic data, we hypothesise that pairs of adult male *Sousa* form at least temporary coalitions or alliances. The use of objects in sexual displays by non-human mammals is rare and, moreover, cooperation between males in the pursuit of an indivisible resource is an evolutionary hurdle relatively few species have overcome. These findings suggest a hitherto unrecognised level of social complexity in humpback dolphins.

## Introduction

While complex male sexual displays are widespread in the animal kingdom^1^, those enriched by some form of object carrying or manipulation are far less prevalent. Notable avian performers include male great bowerbirds (*Ptilonorhynchus nuchalis*), typically holding and/or tossing some of the brightly coloured ornaments used to decorate the courtship arena during their multi-modal sexual displays^2^. Although not directly related to mating success, these components of the displays may increase signal efficacy in attracting and holding the female’s attention^2^. Black wheatears (*Oenanthe leucura*) carry large numbers of heavy stones in flight as a display of mate quality, allowing females to adjust their reproductive effort as a function of the parental and/or phenotypic quality of the male^3^. Occurring after mating but prior to nesting, stone carrying is thus a rare example of a sexual display performed outside the context of mate acquisition^3^. Male palm cockatoos (*Probosciger aterrimus*) fashion drumsticks from live branches or use seed pods to beat against tree limbs, presenting the first tantalising evidence in a species outside our own of tool manufacture and use to serve a socio-sexual purpose^4,5^, rather than one of foraging, as it does in other birds^6^. The phenomenon is particularly rare in non-human mammals. Object carrying by Amazon river dolphins (*Inia geoffrensis*) is deemed a socio-sexual display, although the objects were variable, including plant matter, stones and clay, and some adult females and juveniles also engaged in the behaviour^7^.

Also unusual in the sexual context is the formation of competitive coalitions or alliances among males, particularly for the purpose of access to females. This behaviour is intriguing because individuals cooperate in the pursuit and defence of an indivisible resource^8^, as conceptions cannot be shared. In the scope of mammalian behaviour, such male alliances are uncommon, but well documented in, for example, lions (*Panthera leo*)^9^, red howler monkeys (*Alouatta seniculus*)^10^, Camargue horses (*Equus caballus*)^11^ and Guinea baboons (*Papio papio*)^12^. Male alliance formation has also been documented in the marine realm in several bottlenose dolphin (*Tursiops* spp.) populations^13–15^. One such population, Indo-Pacific bottlenose dolphins (*T. aduncus*) in Shark Bay, Western Australia, exhibits the most complex alliance structure known outside of humans, in which ‘alliances of alliances’ within an open fission-fusion network may remain stable over decades^16–18^.

**Figure 1:**
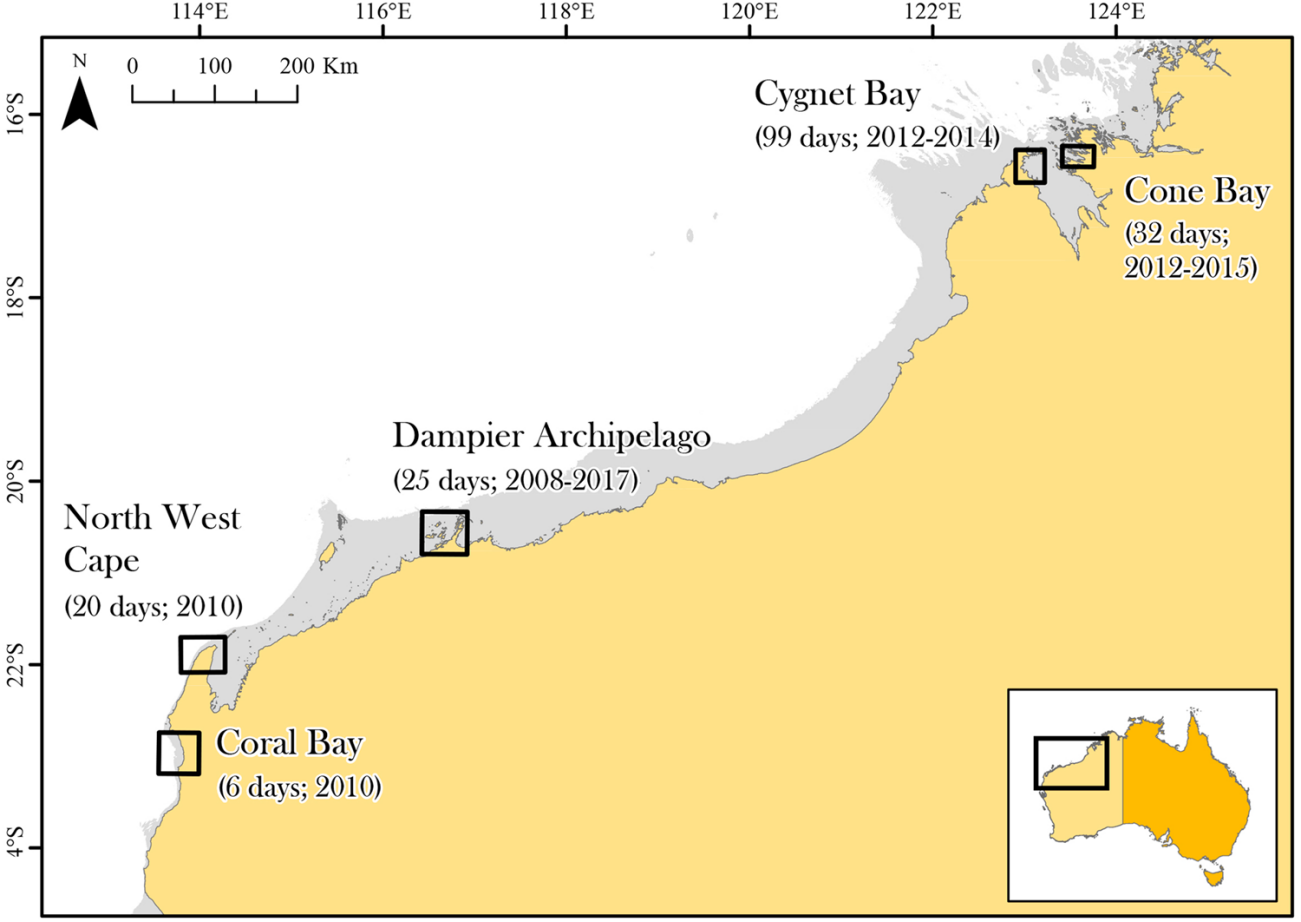
Field sites across north-western Australia at which *Sousa* were observed presenting large marine sponges; Overview of survey effort given in survey days, or part there-of, on the water at each site and over which years in order to illustrate the relative rarity of the behaviour. Waters ≤40 m depth, approximating the range of *Sousa* in north-western Australian waters, are shaded grey. This figure was generated in ArcMap v.10.4 (http://desktop.arcgis.com/en/arcmap/).

The recently classified Australian humpback dolphin (*Sousa sahulensis*, “*Sousa*” hereafter) is found north of ca. 28°S in the shallow, near-shore waters of the northern Australian and southern Papua New Guinean coastlines^19–21^. The social dynamics of the Genus are characterised by a fission-fusion grouping pattern^22,23^, and Australian *Sousa* occur in small populations (<200 individuals, typically far fewer) that are patchily distributed across the species’ range^24–28^. To our knowledge, sexual segregation has not been reported for any of the four *Sousa* species currently recognised. Here, observations of sponge presenting and associated behavioural posturing accumulated over a decade of field research from five separate study sites in Western Australia (Fig. 1) were interrogated with a view to understanding their function. Additionally, we combine behavioural and genetic data to report on the occurrence of adult male *Sousa* associating in pairs.

## Results

### Sponge presenting and physical posturing

Data were collected across a ca. 1,500 km stretch of the tropical Western Australian coastline (Fig. 1) as part of boat-based research on coastal dolphin population biology between 2008 and 2017^26,29–31^. Individual dolphins were photographically identified based on natural markings and visually assigned into one of three age categories (adult, juvenile or calf). Sex was assigned by: observation of dorsal fin characteristics - adult male *Sousa* exhibit distinctive loss of pigmentation (LOP) on the upper half of the dorsal fin^30^; observation of the genital region; the presence of a dependent calf - for adult females; and/or by genetic sexing of biopsy samples collected using a remote darting system^32^ (see Methods).

**Figure 2:**
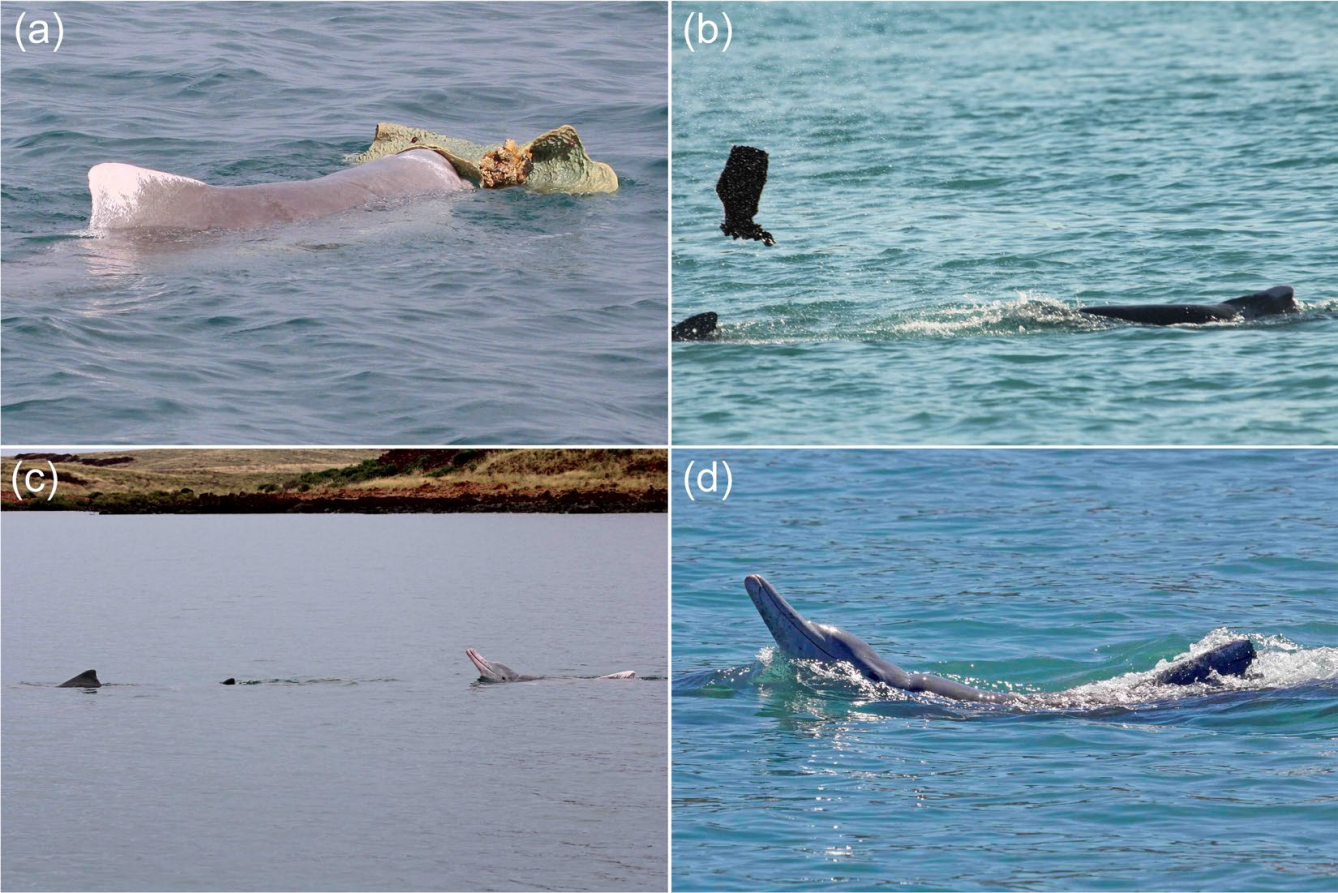
(**a**) Adult male *Sousa* presenting a large marine sponge in proximity to adult females, Cygnet Bay (image credit: F. Smith); (**b**) Adult male *Sousa* tossing a sponge toward an adult female, Cone Bay (image credit: A. Brown); (**c**) Adult male *Sousa* (right of frame) performing “banana pose” in proximity to adult female, Dampier Archipelago (image credit: A. Brown); (**d**) Adult male *Sousa* physically posturing and emitting a trumpeting sound (for ca. 30 seconds) while swimming immediately behind an adult female, Dampier Archipelago (image credit: S. Allen).

We documented *Sousa* presenting large marine sponges on their rostra/melons on 17 occasions (Table 1, Fig. 2a). The mean group size during sponge presenting events was six individuals (range 3–10), and these groups contained calves of weaning age, indicative of the presence of potentially receptive adult females, in 15 of these 17 events (Table 1). By way of comparison, female bottlenose dolphins (*T. aduncus*) are more “attractive” to males when they have a calf of weaning age^33^. At least eight different individuals presented sponges, all of which were adult males (Table 1). On four of these 17 occasions, the adult male *Sousa* with the sponge repeatedly tossed it in the direction of a conspecific (Fig. 2b), three of whom were confirmed female via genetic sexing or the presence of a calf of weaning age.

**Table 1:** Survey date, study site, group composition and individual characteristics from *Sousa* sponge presenting events across north-western Australia.

Survey date	Study site	Group composition	Sponge presenter ID	Sponge presenter sex	Female present
6 Apr 2010	Coral Bay	2 AD, 1 CA	Unk	M^LO^ ^P^	Yes
11 Apr 2010	North West Cape	4 AD	Unk	M^LOP^	Unk
13 Apr 2010	North West Cape	6 AD, 1 CA	SEx11	M^G^	Yes
20 May 2010	Dampier Archipelago	4 AD, 1 CA	SDa12	M^G^	Yes
12 Sep 2012	Cygnet Bay	4 AD, 1 CA	Sc006	M^O^	Yes
21 Sep 2012	Cygnet Bay	2 AD, 1 CA	Sc015	M^G^*	Yes
21 Sep 2012	Cygnet Bay	8 AD, 1 CA	Sc015	M^G^	Yes
25 Sep 2012	Cygnet Bay	6 AD, 1 CA	Sc015	M^G#^	Yes
1 Oct 2012	Cygnet Bay	7 AD, 1 CA	Sc015	M^G^	Yes
21 Oct 2012	Cone Bay	6 AD, 1 CA, 2 JU	Ss008	M^G^	Yes
10 Sep 2013	Cygnet Bay	6 AD, 2 CA	Sc006	M^O^	Yes
16 May 2014	Cygnet Bay	6 AD, 1 CA	Unk	M^LOP^*	Yes
7 Sep 2014	Cone Bay	4 AD, 2 CA	Ss008	M^G#^*	Yes
19 Sep 2014	Cone Bay	2 AD	Ss008	M^G^*	Unk
29 Sep 2015	Cone Bay	5 AD, 2 CA, 3 JU	Ss008	M^G^	Yes
8 Apr 2017	Dampier Archipelago	2 AD, 1 CA, 1 JU	SDa02	M^G#^	Yes
21 Apr 2017	Dampier Archipelago	3 AD, 2 CA	Unk	M^LOP^	Yes

Sex was determined genetically (G), by observation of the genital region (O), or dorsal fin features (LOP). LOP = loss of pigment; AD = adult; JU = juvenile; CA = calf; Unk = unknown. *Sponge tossed toward conspecific; ^#^Banana pose observed.

We also documented three instances of physical posturing prior to or after sponge presenting in which the adult male appeared to flex, with rostrum, head and, once, tail above the water surface (‘banana poses’; Table 1; Fig. 2c). Further, there were three occasions on which an adult male (not presenting a sponge) performed a banana pose directly beside or behind an adult female whilst also producing a ‘trumpeting’ sound from its blowhole (Fig. 2d).

### Associations and re-sights of adult male pairs

**Figure 3:**
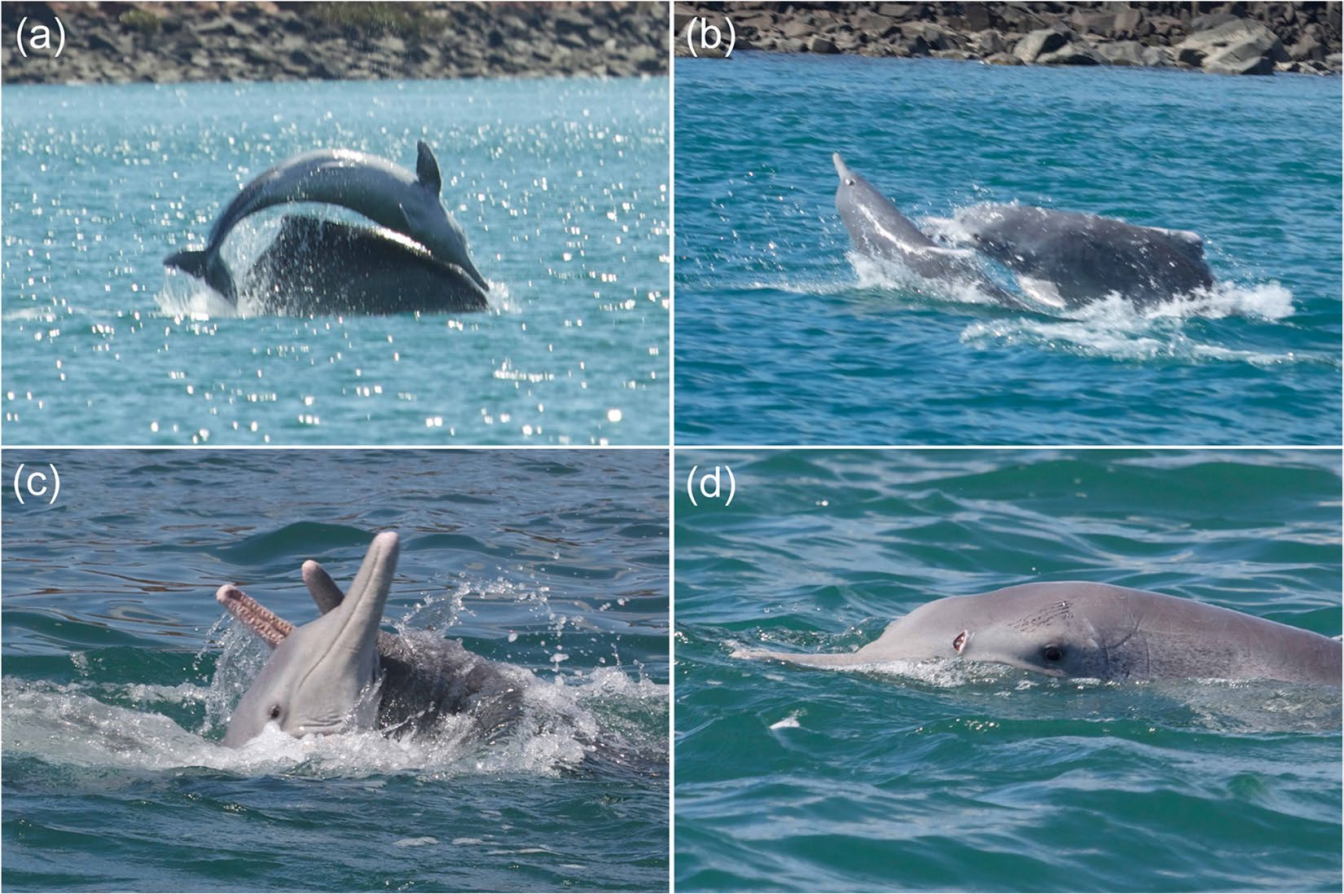
(**a**) One member of a closely associated pair of adult male *Sousa* in the Dampier Archipelago charged a juvenile male, thrusting it into the air; (**b**) The pair flanked the juvenile, one charging, jaw agape; (**c**) The adult male *Sousa* repeatedly corralled and bit the juvenile, while it repeatedly surfaced vertically out of the water in an apparent attempt to avoid harassment, whistling audibly; (**d**) The juvenile *Sousa* with fresh wounds from the teeth of the adult males (image credits: S. Allen).

Of 63 *Sousa* group encounters across north-western Australia in 2010, eleven groups consisted of, or included, pairs of large, adult *Sousa*. All individuals in these pairs were tentatively assigned a sex of male in the field based on their morphology (size and dorsal fin LOP) and behaviour (e.g. coordination in approaching females or aggression toward a conspecific – see Discussion and Fig. 3). All individuals in these pairs were photographically identified with the characteristic dorsal fin LOP of adult males; and all biopsy sampled individuals (n = 13) in these pairs (n = 3 pairs with both members sampled; n = 7 pairs with one individual sampled) were confirmed male by genetic sexing. Repeat visits to three of the five study sites also resulted in the photographic ‘recapture’, or re-sighting, of five of these closely associated pairs of adult male *Sousa* in the same group (Table 2).

**Table 2:** Study sites, dolphin identities, month of initial photo-identification and subsequent re-sight (re-sightings on the same day excluded), and number of occasions on which adult male *Sousa* pairs in each population were documented together (as a fraction of the total number of times each individual was seen).

Study site	Sousa pair identity	1^st^ survey date	Re-sight date	# Times seen together/overall
Dampier Archipelago	SDa21/S21al	Nov 2008	Apr 2011	2/2
Dampier Archipelago	SDa12/S12al	May 2010	Apr 2011	2/2
Cygnet Bay	Sc005/Sc006	Apr 2012	May 2014*	12/20
Cone Bay	Ss008/Ss009	Sep 2014	Sep 2015	4/11
Cone Bay	Ss011/Ss012	Sep 2014	Sep 2015	3/4

*See social network analysis based on associations over a two-year sampling period in Results text.

Four repeated, systematic data collection efforts over two years (2012–2013) at one site (Cygnet Bay, Fig. 1) permitted the generation of association indices between pairs of individuals in that population (see Methods for further details). Social network analysis revealed an overall mean association index of 0.08 (1,000 bootstraps: *SE* = 0.03) for the Cygnet Bay *Sousa* population, including the zeros of no associations (two individuals never seen together). When considering only non-zero associations, a more conservative measure of the overall mean association index, the result was 0.17 (1,000 bootstraps: *SE* = 0.07). The only *Sousa* pairs with high association indices (>0.5) were four mother-calf pairs, with an average index of 0.8 (1,000 bootstraps: *SE* = 0.064), and a pair of adult male *Sousa* (Sc005/Sc006, Table 2), with an association index of 0.61 (1,000 bootstraps: *SE* = 0.11).

## Discussion

We report on multi-modal sexual displays involving object presentation by males in a non-human mammal. Some male *Sousa* present marine sponges and engage in physical posturing and acoustic displays. Our data suggest that marine sponge presenting in *Sousa* is part of a sexual display rather than, for example, a form of object play or foraging. As in other social mammals, object play is common in many dolphin species and has been reported in captivity and in the wild, across age and sex classes, including the carrying and exchanging of items such as seagrass, sea cucumbers, branches, rocks, shells, coral and debris of anthropogenic origins (e.g., plastic bags, bricks and glass bottles)^34–36^. However, given the specificity of object presenting by adult male *Sousa*, i.e. exclusively marine sponges, always by adult males and apparently directed at potentially receptive adult females, object play is not the most parsimonious explanation.

There are four other cases in which object carrying has been reasoned different from play in dolphins: (*i*) ‘shelling’, in which some Indo-Pacific bottlenose dolphins (*T. aduncus*) of both sexes in Shark Bay, Western Australia, carry large gastropod - trumpet (*Syrinx aruanus*) and baler (*Melo amphora*) - shells to the surface in order to extract forage prey that have sought refuge there-in^37^; (*ii*) ‘sponging’, in which particular matrilines of bottlenose dolphins, again, of both sexes in Shark Bay, use marine sponges as foraging tools^38–42^; (*iii*) the carrying or thrashing of plant matter, stones, clay and other objects primarily by adult males, deemed likely to represent a socio-sexual display in Amazon river dolphins of Brazil^7^; and (*iv*) a sponge carrying event by an adult male *Sousa* in Queensland, Australia^43^, which was interpreted as foraging involving tool use (although this may warrant re-visiting in light of our findings).

Some of the attributes of the *Sousa* encounter described in detail in (*iv*) above^43^ bear resemblance to those documented here, i.e. it was only an adult male that carried the sponge, he moved from one group to another, and the individual was always within the vicinity of other adults, some of which were female. Sponges may indeed serve more than one purpose, but without further supporting evidence, that account remains enigmatic. We can discount the use of sponges as foraging tools by north-western Australian *Sousa* from our data with confidence for several reasons. First, we report on multiple instances of sponge presentation and/or tossing, all of which involved adult males presenting the sponge. This is in stark contrast to the previously described sponge carrying behaviour for the purpose of foraging in Shark Bay dolphins, which, although engaged in by some males, is heavily female-biased and exclusively linked to foraging^38,42^. Second, the ‘presenter’ approached and/or followed likely receptive adult females in most instances of sponge presenting. Third, behavioural posturing sometimes preceded or succeeded sponge presentation, none of which is associated with foraging behaviour. Fourth, the sponge presenting events by *Sousa* reported here bore none of the stereotypical characteristics of the foraging behaviour exhibited by sponging or shelling Indo-Pacific bottlenose dolphins in Shark Bay^37,38^.

Object carrying by Amazon river dolphins is likely to be a sexual display performed predominantly by adult males, although the objects were many and varied, and 25% of events by identifiable individuals were by adult females and/or juveniles^7^. While not as expansive a dataset on *Sousa*, our signal is strong. Moreover, the behavioural posturing by male *Sousa* is similar to that exhibited by bottlenose dolphins engaged in sexual displays in Shark Bay^16^. The “rooster strut”, for example, is performed by individual males or simultaneously by pairs of males in Shark Bay, where the head is arched above the surface and bobbed up and down, usually in the presence of a female^16^. Sponge presentation in *Sousa* thus forms part of multi-modal sexual displays that may have evolved to attract the female’s attention. Given that the sponge is an adornment easily seen or detected via echolocation, we hypothesize that this component may serve as an honest signal for male quality. Large marine sponges are often firmly attached to hard substrates^44^, particularly where they must endure extreme tidal movements (such as those typical of north-western Australia), and many contain chemical defences to prevent overgrowth or predation^45^. Sponges may therefore require dexterity and strength to remove, while conceivably exposing the dolphin to both discomfort from chemical defences and greater risk of shark attack while otherwise engaged^46^. Obtaining and presenting the sponge may also represent a signal of cognitive ability, thereby indirectly indicating male quality where higher cognitive performance is linked to male mating success^2,47^. Alternatively, and perhaps more likely, the presentation and, in particular, tossing of the large sponges in the direction of the female may serve to intimidate, analogous to branch dragging and shaking or the hurling of rocks by male chimpanzees (*Pan troglodytes*) to enhance their charging displays during sexual coercion^48,49^. Indeed, a threatening or dominance aspect to such displays might also explain the thrashing of plant material and other objects by Amazon river dolphins^7^.

Some pairs of visually and/or genetically confirmed adult male *Sousa* were also: frequently seen in close association; photographically recaptured together over months and years; and engaged in apparently coordinated approaches to conspecifics that were both affiliative and aggressive in nature. For instance, a closely associated pair of adult males (Ss008/Ss009, Table 2) made a directed approach to an adult female with a calf of weaning age, leaping synchronously from ≈200 m away before positioning themselves behind the mother-calf pair. They proceeded to follow them, before Ss008 performed a banana pose and, later, presented and then tossed a sponge toward the female (Fig. 2b). Another pair of adult male *Sousa* (SDa07/SDa08, both biopsy sampled when travelling together in the Dampier Archipelago eleven days earlier) displayed coordinated, overt aggression toward a juvenile male *Sousa* that had approached a small subgroup of mother-calf pairs in the vicinity immediately prior to this interaction (Fig. 3).

Furthermore, one pair in a small population at Cygnet Bay, one member of which was documented presenting sponges in the presence of potentially receptive females, associated at levels that would qualify them as allied. Although the evidence we present here on putative coalition or alliance formation in adult male *Sousa* is preliminary only, there are intriguing parallels with those of the well-documented male bottlenose dolphin alliances in Shark Bay, who work together in pairs and trios to sequester and control the movements of individual oestrous females^17^. Long-term field research in Shark Bay has revealed the occurrence of adult males that are repeatedly sighted together (over months and years), that frequently approach females and engage in sexual displays, and that exhibit coordinated, directed aggression toward conspecifics on occasion^17,50^. Since these animals are “cooperating to compete”^8^, future research on *Sousa* should aim to quantify the stability and durability of male-male associations, if these associations are indeed cooperative in nature^51^, whether both members of male pairs perform multi-modal sexual displays, including sponge presenting, and whether or not these factors influence paternity success.

Taken together, these findings suggest a hitherto unrecognized level of social complexity in Australian *Sousa*. Despite their vastly different evolutionary histories, some cetacean species appear to have converged on similar complexity and flexibility in behaviour and social systems as some of the more cognitively advanced bird and great ape species, including our own.

## Methods

Field research was conducted from small (5–6 m) boats at each of five research sites across a ca. 1,500 km stretch of the north-western Australian coastline between 2008 and 2017 (Fig. 1). Our efforts were concentrated in the austral dry seasons (April through October) in order to avoid tropical cyclones. Weather-dependent (no rain, low wind and swell) surveys were carried out as part of several broader research programs on coastal dolphin population biology^19,26,29–31^. Teams of 3–5 observers searched shallow (<40 m deep) coastal waters and recorded the following data on encountering dolphins: species, group size, group composition and behaviour. A ‘group’ was defined as any individual surfacing within ca. 100 m of another individual and engaged in similar behaviour.

Individual dolphins were photographically identified based on natural markings on the dorsal fin^52^, and visually assigned into one of three age categories (adult, juvenile or calf). Dependent calves were defined as ≤½ the length of, and consistently observed in close association (≤10 m) with, their mothers; juveniles were defined as approximately 1/2–2/3 the length of an adult and not consistently associated with an adult. The sex of individuals was assigned where possible, either by observation of dorsal fin characteristics (see below) and/or the genital region, by the presence of a dependent calf (for adult females), and/or later confirmed by genetic sexing of biopsy samples collected using a remote sampling system^32^. Sexual dimorphism is apparent in adult Australian *Sousa*, males showing a distinctive loss of pigmentation (LOP) on the upper half of the dorsal fin, such that their sex can be predicted based on analyses of dorsal fin images with ≥93% accuracy^30^. Genetic sexing was carried out as per the methods outlined in Gilson *et al*.^53^.

We carried out repeat visits to three of the five field sites where sponge presenting was observed, such that some individuals and closely associated pairs of individuals were photographically ‘recaptured’ over time. Systematic, standardised transects were completed at two of these sites, but only one (Cygnet Bay) was sampled sufficiently enough (four repeated data collection efforts in 2012 and 2013, see Brown *et al*.^26^) to generate association indices between pairs of individuals within the population. Hence, some opportunistic matches were made between original and subsequent surveys of *Sousa* groups, but only the photo-identification data from Cygnet Bay was used to calculate association indices, using the simple ratio index (SRI), in SOCPROG 2.7^54^. The SRI is an estimate of the proportion of time two animals spend together (0 for pairs of animals never documented associating; 1 for pairs always seen together) and is the most appropriate measure for defining association by presence in the same group^55^. Animals identified in the same group on a given survey day (sampling period) were considered associated. Any sightings in which ≥50% of the individuals in the group were not identified were discarded. In accordance with prior research on coastal delphinids^56^, only animals sighted on ≥5 occasions were used in the analysis.

All field and laboratory techniques adhered to long-established standards for small cetacean research^32,52–54^.

### Data availability statement

The datasets generated during and/or analysed during the current study are available from the corresponding author on reasonable request.

### Approvals

This research was conducted under permits for the scientific use of animals from the Western Australian (WA) Department of Parks and Wildlife, and a firearms licence from the WA Police. The Murdoch University Animal Ethics Committee approved all experimental protocols and the research was carried out following consultation with Murujuga, Bardi Jawi and Dambimangari traditional owners.

### Accordance

All research was carried out in accordance with the relevant guidelines and regulations.

### Third party rights

SJA and AMB took all the images in Figs 2 and 3, except for 2(a), taken by F. Smith, as per the acknowledgements. We sought and gained explicit permission to use the image.
